# Community Psychosis Risk Screening: An Instrument Development Investigation

**DOI:** 10.20900/jpbs.20200019

**Published:** 2020-08-20

**Authors:** Lauren M. Ellman, Jason Schiffman, Vijay A. Mittal

**Affiliations:** 1 Department of Psychology, Temple University, Philadelphia, 19122, PA, USA; 2 Department of Psychology, University of Maryland-Baltimore County, Baltimore, 21228, MD, USA; 3 Department of Psychological Science, University of California, Irvine, Irvine, 92697, CA, USA; 4 Departments of Psychology and Psychiatry and Behavioral Sciences, Northwestern University, Evanston, 60208, IL, USA

**Keywords:** clinical high risk, psychosis, schizophrenia, prodrome, risk screening, psychosis-risk questionnaire

## Abstract

Schizophrenia and other psychotic disorders are serious psychiatric disorders that are associated with substantial societal, family, and individual costs/distress. Evidence suggests that early intervention can improve prognostic outcomes; therefore, it is essential to accurately identify those at risk for psychosis before full psychotic symptoms emerge. The purpose of our study is to develop a brief, valid screening questionnaire to identify individuals at risk for psychosis in non-clinical populations across 3 large, community catchment areas with diverse populations. This is a needed study, as the current screening tools for at-risk psychotic populations in the US have been validated only in clinical and/or treatment seeking samples, which are not likely to generalize beyond these specialized settings. The specific aims are as follows: (1) to determine norms and prevalence rates of attenuated positive psychotic symptoms across 3 diverse, community catchment areas and (2) to develop a screening questionnaire, inclusive of both symptom-based and risk factor-based questions. Our study will develop an essential screening tool that will identify which individuals have the greatest need of follow-up with structured interviews in both research and clinical settings. Our study has the potential for major contributions to the early detection and prevention of psychotic disorders.

## INTRODUCTION

Longer duration of untreated psychosis has been repeatedly associated with more severe courses of psychosis [1–5], creating an urgency for the detection and identification of individuals in the early phases of psychosis, including the at-risk period of illness. It is now clear that a period characterized by functional decline and subthreshold psychotic symptoms exists prior to the onset of a full-blown psychotic disorders, such as schizophrenia [5]. This period is termed the *prodrome* of psychosis and can range in duration from brief to approximately 5 years, with the majority of cases developing a psychotic disorder within 1 year [6–9]. Prodromal signs include changes in perceptual and thought processes within the past year, such as hearing things that others do not hear without clear conviction that the experiences occurred [5]. Previous studies of clinically-referred samples (i.e., those seeking treatment), in which high-risk clinical states were empirically defined as a recent onset or worsening of subsyndromal psychotic symptoms (termed clinical high risk for psychosis or CHR), have reported conversion rates to psychotic disorders of 9% to 76% across 1- to 9-year follow-up intervals, with most large-scale studies reporting conversion rates of 20–40% [9,10]. Clinical interviews such as the Structured Interview for Psychosis-Risk Syndromes (SIPS) remain the gold standard for accurate identification of those at risk for psychosis, however, this approach is time intensive, requires extensive clinical training, and tends to be effective only among clinical populations [11,12]. Questionnaire-based methods of detecting individuals at risk for psychosis offer advantages to complement interview approaches, with the promise of facilitating more accurate and widespread identification that could have implications for the reduction of duration of untreated psychosis. Self-report approaches to identification of those at risk for psychosis have been investigated in a series of studies and demonstrate good validity and promising positive predictive values (PPVs) when compared with semi-structured interviews [13,14]. Although there are findings from Europe of successful community psychosis risk screening [15,16], nearly all of the existing screening tool-to-interview validation studies have used treatment seeking and/or clinical high risk (CHR) for psychosis samples, carefully selected and recruited for genetic risk or suspected clinical risk, through referrals or preliminary phone screenings [6]. Thus, valid and effective early screening in non-clinical US samples is needed. Further motivating this work, the vast majority of those who develop psychotic disorders do not seek treatment until well after the onset of a psychotic disorder suggesting a need for broader outreach [1–3].

The preponderance of existing screening tools are based solely on report of attenuated positive psychotic symptoms (APPS), such as perceptual abnormalities, paranoid ideation, etc. [13]. Although these symptoms are primarily used to determine CHR for psychosis status with semi-structured interviews, they are very common in the population and may not be sufficiently specific to differentiate CHR from psychosis status from non-clinical APPS in a questionnaire format [17–19]. More importantly, there now is increasing evidence that positive-symptom-only screening tools perform sub-optimally in non-clinical samples [20] (see “Preliminary Studies”). In part, this is likely due to unmeasured variables that improve performance of these screening tools in CHR specialty clinics (e.g., clients who connect with specialty clinics are effectively “prescreened”, given that they have either self-identified or been identified by someone else as being at risk for psychosis). Additionally, there are a number of other symptom domains and risk factors that are commonly found in both the prodromal and premorbid periods of psychosis, that likely can improve prediction of CHR for psychosis when assessed along with APPS (see Preliminary Studies) and have been demonstrated to improve prediction of conversion to psychosis in “risk-calculator studies” (despite the fact that conversion is solely based on psychotic-level positive symptoms) [9,11,21,22]. While our study will not examine conversion to psychosis, the aforementioned findings indicate that prediction can be substantially improved using diverse symptom measures combined with APPS.

Our collaborative R-01, named the Multi-site Assessment of Psychosis-risk (MAP) study, will address the aforementioned issues by developing a screening tool to identify individuals at CHR for psychosis in a sample of adolescents/young adults (aged 16–30 years) drawn from 3 large, US community catchment areas. This screening tool will incorporate APPS items from 3 widely used psychosis screening questionnaires (See Psychosis-Risk Measures) and non-APPS and risk factor-based measures that are associated with psychosis risk.

### Inclusion of Non-APPS Clinical Measures

Multiple non-APPS clinical domains have been repeatedly found in the premorbid and prodromal periods of psychotic disorders and therefore may hold potential to improve prediction of who may be at CHR. For instance, symptoms of depression, anxiety, social anxiety, and dissociative experiences all have been found to occur in the prodrome of psychotic disorders [23–27]. Further, other clinical correlates, such as reduced hedonic functioning (particularly for anticipatory pleasure), and negative symptoms, are not only representative of the psychosis prodrome, but are cardinal clinical symptoms of psychotic disorders [23,28–34]. These constructs have not been the primary focus of risk screening, likely because of their smaller effect sizes relative to APPS [13]. Nonetheless, including these established precursors may increase potential to predict risk, and screening questionnaires that incorporate both APPS and non-APPS information may facilitate more accurate prediction than APPS alone. The MAP study will examine constructs that tap into both categories and, thereby, be positioned to develop an effective general population screening tool (Aim 2).

### Inclusion of Risk Factor-Based Measures

A number of constructs that can be reliably measured via questionnaire are associated with risk for psychosis, but have not yet been incorporated in psychosis-risk screening questionnaires, including: genetic high risk for psychotic and related disorders, decreases in social and role functioning, moderate-to-heavy substance use (e.g., cannabis use), a history of traumatic life events, increases in perceived stress, perceived racial discrimination, immigrant status, race/ethnicity (e.g., African American status), familial stress, and increased sleep disturbance [9,35–45]. The MAP study will be the first to incorporate a number of primary risk factors for psychotic disorders into the development of a psychosis-risk screening tool. Based on preliminary findings, we anticipate that the combination of APPS, non-APPS, and risk factor-based measures will maximize our ability to use self-report information to predict psychosis risk (Aim 2, see Improving prediction of CHR for psychosis using questionnaires).

### SVariation in APPS in the United States (US)

Although a number of studies outside the US have examined prevalence rates of APPS in the general population (particularly in Europe) and have linked these symptoms to many of the primary risk factors for psychosis, only one study in the US has established normed prevalence rates of APPS in non-clinical populations and this study was based on younger participants [46,47]. Similarly, no study in the US has determined whether prevalence of APPS varies in multiple diverse samples by demographic factors, such as immigrant status, which has been repeatedly found in European samples [48]. Determining the rate of APPS in risk questionnaires in non-clinical samples in the US is vital to psychosis-risk assessment, given that the primary psychosis-risk assessment measures have only been examined in clinical samples. The MAP study will also allow us to determine whether European findings are generalizable to US populations (Aim 1).

## INNOVATION

This is the first study to attempt to develop a questionnaire to identify individuals at risk for psychosis in a non-clinical US sample incorporating both symptom and risk-factor based items. Although many of these constructs have been associated with only incremental increases in psychosis risk, this study will rely on algorithms that combine constructs to predict overlapping and non-overlapping groups of individuals at CHR for psychosis. Not only is this an innovative approach to detecting those at potential risk for psychosis (as the preponderance of studies solely focus on APPS for screening purposes), but this strategy is more consonant with current concepts of psychopathology that suggest that there likely are multiple pathways to the same clinical outcomes (i.e., equifinality). The MAP study also will be the first to establish norms for and demographic correlates of APPS across multiple, diverse, US, catchment areas [46]. This aim (Aim 1) is aligned with NIMH priorities to examine psychopathology and risk on continuums (e.g., RDoC) [49,50], and findings from this aim could substantially enhance efforts to examine psychosis on a dimensional scale.

### The Potential Impact

The proposed study has the potential to substantially influence strategies for early detection of individuals at risk for psychosis in the general US population. The questionnaire developed by this study could be used to determine who might benefit from additional assessments in treatment settings and in a variety of other community settings (e.g., schools, medical settings). It is important to note that this tool would not be meant to replace detailed clinical assessments, but rather to improve detection of individuals likely to have the greatest need for clinical and/or research follow-up. With the addition of Attenuated Psychosis Syndrome (APS) as a condition for further study in the DSM-5, along with Other Specified Schizophrenia Spectrum and Other Psychotic Disorder which includes APS, valid screening of suspected APS may become important for practitioners with little background in psychosis-risk who may be increasingly assessing for APS. This is the time for such a screening tool to be developed, when APS is still a condition for further study, as it is essential to prepare for wide-spread use of the diagnosis. As noted, those who develop psychotic disorders often do not seek treatment until after the onset of psychotic symptoms, and longer duration of untreated psychosis has been repeatedly associated with more severe courses of psychotic disorders, suggesting that a valid early screening tool could be of great benefit to individual and public health outcomes [1–3]. Given that untreated mental illness can also have significant implications for academic, occupational and social outcomes, a valid early screening tool could potentially offer additional long-term benefits for individuals and the public [51–53]. Further, even those at CHR for psychosis who do not convert to psychosis often experience other adverse mental health outcomes; therefore, focusing on predicting CHR accurately is an extremely important clinical area, irrespective of conversion outcomes [54–57]. Finally, a valid screening questionnaire could also provide a useful tool for recruitment of CHR samples for both basic and applied research. Such studies typically administer the SIPS to determine risk status and eligibility for study participation, which can be resource-intensive; therefore, effective screening could maximize assessors’ time by identifying individuals most likely to be true positives on the SIPS. Overall, the MAP study could have a substantial impact on mental and public health initiatives for early detection of severe mental disorders.

## PRELIMINARY STUDIES

Each collaborating site has published extensively on topics relevant to the study aims and is among only a handful of US sites examining risk factors for psychosis in non-clinical samples. Our pilot data focused primarily on non-clinical, undergraduate samples (as well as some community volunteers); as a result, reported estimates may be conservative, in that college populations could be self-selected to have less frequent and less severe serious mental health issues than the broader community.

### Prevalence of APPS Symptoms in Non-Clinical Populations

At the Temple University (TU) site, the prevalence of participants scoring above the established Prodromal Questionnaire (PQ) cut-off (endorsing 8 or more distressing positive symptoms; PQhigh) is 13.69% out of 724 undergraduate students [58]. This rate is lower than 17.9% found in a non-clinical undergraduate sample comprised primarily of ethnic minorities from City College in New York [17], suggesting that demographic factors (e.g., race/ethnicity) may influence APPS rates, as has been found repeatedly in European samples [59–61]. Further, in the TU sample there were a number of APPS items that were commonly endorsed and, therefore, may not be predictive of CHR status in this population [58]. Interestingly, some of these commonly endorsed items have been retained in the brief version of the PQ (PQ-B), which excludes items that were commonly endorsed in the PQ’s original sample of UCLA undergraduates, suggesting variability in item-level APPS responding by site [62,63]. To account for this potential variability, the MAP study will use the original PQ instead of the PQ-B (as all PQ-B items overlap with the PQ, allowing both versions to be examined). Further, these results reinforce the need not only to develop APPS norms across multiple samples, but also to determine the correlates of item-level endorsement of APPS across sites. We estimate that 13% of our sample will score above at least one of 2 psychosis screening measure cut-offs, based on the preliminary results found with the PQ screening measure alone.

### Convergent and Discriminant Validity of CHR Screening Measures

At the UMBC site, 3 CHR screening measures (PQ-B, Prime Screen, and Youth Psychosis At-Risk Questionnaire-Brief) have demonstrated a pattern of convergent validity with each other (*r* = 0.61–0.77), discriminant validity with measures of other constructs (e.g., Beck Depression Inventory, *r* = 0.20–0.52), and high test-retest reliability (*r* = 0.78–0.82) [64]. Similarly, the TU site found that APPS items on the PQ had discriminant validity with the Social Phobia Scale (SPS), loading onto different factors, despite being correlated constructs (*r* = 0.44 to 0.48, SPS with APPS subscales) [65]. These findings indicate an established record of examining psychometrics of psychosis-risk screening tools and strongly suggest that non-psychosis measures contribute unique variance to risk prediction.

### Criterion Validity

The UMBC site administered psychosis-risk screens prior to full SIPS interviews in a sample of 48 services-seeking youth, many at CHR for psychosis. The screening measures demonstrated strong predictive properties in comparison to the SIPS, with predictive accuracy between .69 and 0.80 [14]. In contrast, in non-clinical TU undergraduates—using established cut-off criteria for the PQ (PQhigh) and both 3 or fewer distressing APPS and 8 or fewer total APPS for the lower risk group (PQlow; cut points equivalent to the means of the sample) [66]—initial results found that 18.57% (13 out of 70) of the PQhigh group met SIPS criteria for CHR for psychosis. Conversely, 3.9% (2 out of 51) of the PQlow group met SIPS criteria for CHR for psychosis. Collectively, these studies suggest that more work in non-clinical samples is needed to create effective APPS screening tools for the general population.

### Risk Factors Potentially Associated with CHR for Psychosis in Non-Clinical Populations

First, a number of symptom domains outside of psychotic symptoms have been consistently associated with increases in APPS and with risk of crossing the cut-off scores for psychosis-risk screening measures. Specifically, increases in co-occurring symptoms found in CHR and psychosis populations, such as social anxiety, generalized anxiety, depressive symptoms, and decreases in anticipatory (but not consummatory) pleasure all have been associated with increases in APPS, as well as with potentially higher risk for psychotic disorders (based on questionnaire cut-offs) [19,65,67]. Second, a number of risk factors that have been found in CHR and psychotic populations have also been associated with increases in APPS and increased odds of crossing psychosis-risk screening questionnaire cut-offs. Specifically, family history of substance use, family history of mood/anxiety symptoms, and family history of psychotic symptoms were related to a higher likelihood of college students at TU being classified as PQhigh [58]. Further, a history of trauma, moderate-to-heavy cannabis use, living in low ethnic density neighborhoods (only for Black participants), increases in perceived racial discrimination, low social functioning and increases in perceived stress all have been associated with increases in APPS and increased odds of being classified as PQhigh in a series of studies using TU, City College of New York (a collaborating site with TU), and UMBC undergraduates [17–19,68,69]. Similarly, the Northwestern site has a number of findings in non-clinical undergraduate populations, including (but not limited to) increases in movement abnormalities, specific genetic polymorphisms, and fronto-striatal dysfunction being linked to increases in APPS [70–74]. Cumulatively, these findings suggest that many of the risk factors that have been associated with CHR for psychosis also occur in non-clinical populations. This body of work also supports the feasibility of the MAP study, highlighting that each study site has published extensively on risk factors for psychosis in non-clinical populations.

### Improving Prediction of CHR for Psychosis Using Questionnaires

**Figure 1.**
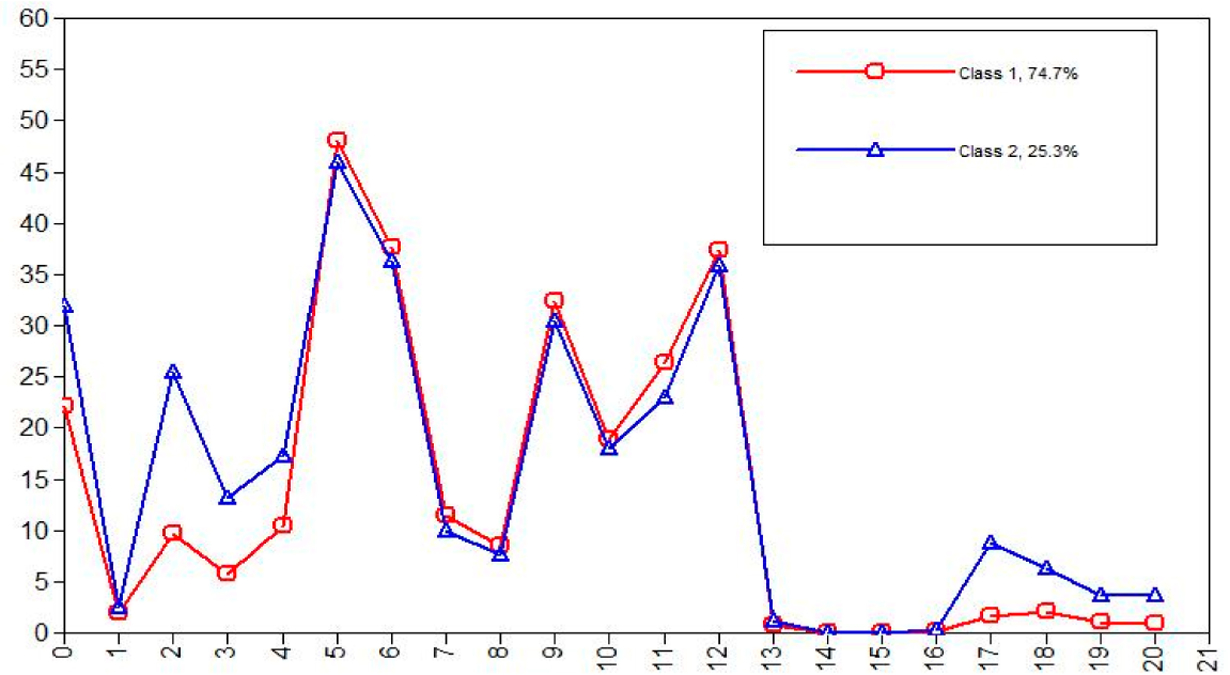
LCA Profiles depicts LCA means for the two classes on y-axis (*N* = 2836). The x-axis categories are the following (abbreviations and descriptions can be found in s5a “Self-report Measures”): 0. PSS 1. LEC 2. SPS 3. CESD 4. STAI-Trait 5 & 6 TEPS (anticipatory and consummatory) 7–12 SFS subdomains 13. Cannabis use 14. Opiod use 15. Amphetamine Use 16. PSQI 17.PQ-Unusual thought content 18. PQ-Paranoia 19. PQ-Perceptual Disturbances 20. PQ-Disorganization.

A series of questionnaires were administered to 2836 TU, nonclinical undergraduates, and then a portion of the PQhigh and PQlow groups were followed up with the SIPS (51 PQlow and 70 PQhigh). A two-class latent class analysis (LCA) model best fit the data (Figure 1). Class 2 was elevated on a number of variables associated with psychosis (e.g., depression, anxiety, perceived stress, and subdomains of the PQ, etc.). Being in class 2 was associated with a 14.54 increased odds of being at CHR for psychosis (*p* = 0.001) compared with a 5.59 odds using the PQ cut-off alone (*p* = 0.028). Further, 3 permutations were created to determine whether adding variables to the PQ could improve prediction of those at CHR for psychosis: 1. PQ cut-off plus a broad familial high risk variable (Fhx; chosen based on previously preliminary data, not included in LCA), including any family history of mood/anxiety or psychosis symptoms in first through third degree relatives (which was endorsed by 57.93% of participants and is a broader definition of familial risk than any CHR study), 2. High unusual thought content (UTC) and elevations on perceived stress, depression, generalized anxiety, or social phobia (using the class 2 LCA means as cut-offs) and, 3. UTC and Fhx. As Table 1 indicates, compared to the PQ alone, the 3 permutations of variables substantially improve prediction of CHR for psychosis without any changes in sensitivities or negative predictive values. Specifically, the PQ cut-off was associated with an 18.6% PPV, while combining high UTC (as this domain differentiated the classes more than the other PQ domain) with Fhx improved the PPV more than 10 percentage points to 28.89%. Adding additional variables substantially reduced the false positive rates and substantially increased specificity with no losses to sensitivity. Specifically, the false positive rate was reduced by 56% in the last permutation listed in Table 1 and specificity was improved by 24%, meaning that considerably fewer individuals would need to be screened in order to identify those at CHR. Further, it appears as if these questionnaire risk groups are only partially overlapping, as 10.78% of the full sample were high on the non-APPS LCA clinical symptoms, but reported no family history of mood or psychosis symptoms and 34.87% of the sample reported a family history of mood or psychosis symptoms, but were not elevated on the non-psychosis clinical symptoms. This latter point supports our aim of identifying overlapping and non-overlapping patterns of questionnaire responding among individuals who are at CHR for psychosis. We also will have substantially more power in the MAP study; therefore increasing the likelihood of even higher PPVs than those obtained in these preliminary findings.

## THE APPROACH

The MAP study was funded in July of 2017. This study will use a prospective, longitudinal design across 3 demographically diverse catchment areas. In years 1–4, 12,000 participants will complete an online battery of self-report questionnaires assessing risk factors for psychosis, clinical correlates of psychosis, and 3 psychosis-risk screening questionnaires. Based on established cut-off scores from 2 of these psychosis-risk screening measures, the PQ and the Prime (see Psychosis-Risk Measures; the Prod Screen was not used for selection of participants due to substantial over endorsement of symptoms in pilot testing prior to the study beginning), an estimated 26% of participants (13% questionnaire higher risk-QHR; 13% questionnaire lower risk-QLR) will complete structured diagnostic interviews that assess psychosis-risk syndromes and DSM-5 Psychopathology (2000 interviews total across the 3 sites). These projected sample sizes are based on pilot data in Criterion Validity (P selection is described further in S4d) and accounts for 36% potential loss to follow-up (i.e., 13% of 12,000 participants = 1560 QHR; therefore interviewing 1,000 of these individuals allows for loss-to-follow-up). We also have strategies in place to encourage retention, including incentivizing the in-person interview more than the online battery.

### Participants

Participants will be a socioeconomically, racially, and ethnically diverse sample of male and female adolescents/young adults (ages 16–30) from 3 catchment areas: Philadelphia County (PA), Cook (IL; includes Chicago) and Baltimore City/County (MD). There will be an equal gender distribution (see Table 2 for projected demographic and gender distributions by site).

### Rationale for Participant Selection

Previous studies suggest that our recruitment age range overlaps with the peak age of onset of psychotic disorders (ages 17–35) [23]. Although it is possible that a small minority of participants will meet formal diagnostic criteria for a psychotic disorder, the vast majority of our sample will be within the risk period for psychotic disorders (mean age = 20.5, consistent with recently noted ages of onset between 20’s and mid-30’s) [75]. Comparable to our ongoing studies of youth at CHR, we selected 16 as the lower age limit, as it represents the youngest age CHR for psychosis can be detected without compromising the possible validity of the measure given potential differences in questionnaire responding and neurodevelopment among individuals younger than 16 [76,77]. Further, our sample will be a non-clinical, community sample drawn from flyers and advertisements on websites (e.g., Facebook, Craig’s List, etc.). This method should produce a more diverse sample than random number dialing methods that rely entirely on landlines (especially in this age group) and, therefore, should increase generalizability to same-aged populations.

### Participant Recruitment and Feasibility

In order to obtain a representative sample across the 3 catchment areas, we will recruit our participants from flyers posted around our catchments areas (e.g., coffee shops, community centers, religious meeting places, etc.) and ads on a variety of websites. Interested participants will be asked to complete the baseline questionnaires on the internet, after which they will receive a $10 gift card, with the knowledge that some participants will be selected for follow up (see Interviews and Neurocognitive battery). The target goals for the initial screening portion of the study are feasible, as each site has recruited comparable sample sizes in their ongoing studies of non-clinical populations. There are no exclusion criteria for this portion of the study beyond being within the age range of 16–30. However, at 4-month intervals, the gender, ethnic, and racial distributions of the sample will be examined at each site, and recruitment will be modified accordingly in order to maintain equal gender distributions and racial/ethnic distributions representative of the catchment areas.

### Interviews and Neurocognitive Battery

**Figure 2.**
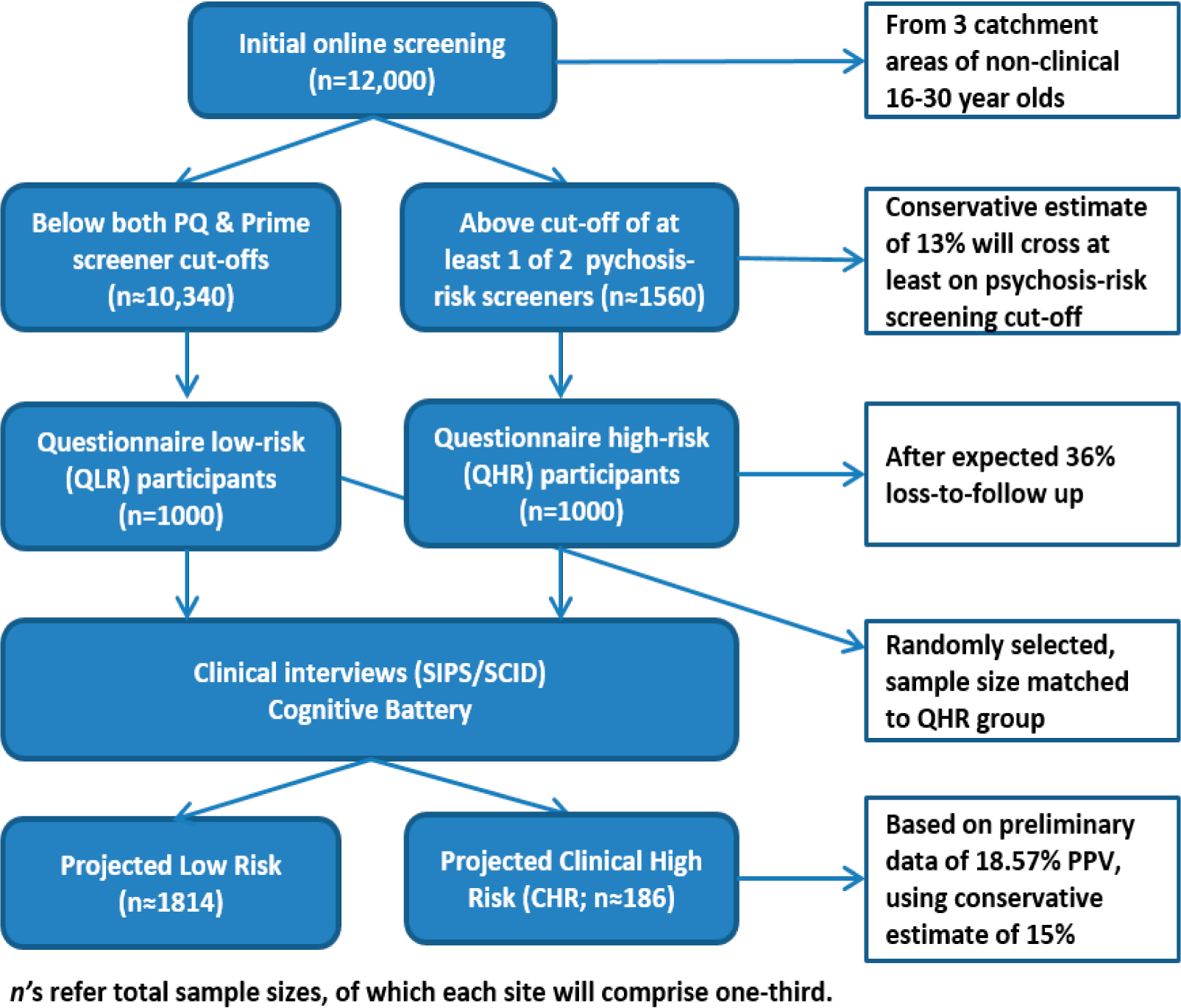
Participant recruitment and sample sizes.

Two-thousand (1000 QHR and 1000 QLR) of study participants will be included in the interview portion of the study based on 2 methods: (1) all of those who cross the established cut-offs of at least 1 of 2 psychosis screening tools (see Psychosis-Risk Measures and Figure 2) will be considered QHR and be contacted for interviews (out of an anticipated pool of 1560), and (2) randomly selected individuals below both psychosis screening measure cut-offs (QLR) will be invited to participate in the interview portions of the study. We anticipate that over 13–18% of each study sample will cross the cut-off of at least one of the 2 psychosis-risk screening measures (13–18% is a low estimate based solely on using 1 screening measure, see Prevalence of APPS symptoms in Non-Clinical Populations); therefore, planning to interview 1000 participants deemed QHR accounts for the possibility of refusal/disinterest in continued participation in the study. The same sample size of QLR participants will be randomly selected from the remaining 85–87% of the sample. To increase retention for the clinical interview portions of the study, participants will receive reminder calls and emails near the day of their scheduled follow-up assessment, as well as $80 for their participation and $20 for travel ($100 total). We also will collect alternate contact information in order to track participants for potential follow-up studies.

### Procedure (See Figure 2)

#### Time 1 (T1)

The initial screening will take place on the internet, following eligibility assessment by callers. Participants will receive a link to complete study consent/assent, as well as a battery of questionnaires (see SELF-REPORT MEASURES (T1 AND T2), Psychosis-Risk Measures, Non-Psychotic Clinical Measures Associated with Psychosis, Risk Factor Measures). All questionnaires will be completed using the online program Qualtrics (Provo, Utah) and will be administered to the entire 12,000 participants at T1, given that T1 responses will determine QHR and QLR groups. Further, this approach will allow for supplementary analyses examining potential contributors to variations in APPS across the sites. This battery should take approximately 45 min based on pilot testing.

#### Time 2 (T2)

Approximately 1 week after T1, QHR and QLR participants will be invited to complete diagnostic interviews (see Semi-Structured Interviews (T2)), with interviewers blind to potential risk status. participants also will complete a brief, computerized cognitive battery, an IQ estimate, and will repeat the self-report questionnaire battery (this session will take an average of 5 h).

## SELF-REPORT MEASURES (T1 AND T2)

Note that some measures (none of the primary measures) were altered in order to reduce participant burden and to remove questions that may indicate safety concerns, given that participants completed the initial phase of questionnaires online prior to meeting study staff.

### 

#### Infrequency/random responding

Given the possibility of careless and fraudulent responding to self-report items, the project will include the embedded infrequency and reliability scales from the Assessment of Depression Inventory [78]. This approach was developed in a validation study examining groups of clinical, feigning populations, and computer-generated random profiles, which found that elevations on either scale detected 94.9% of random responders [79]. These measures include a total of 10 self-report items that will be interspersed throughout the proposed battery [79]. These items were added to determine if participants were paying attention and to determine whether the participant was a bot or engaged in fraudulent responding. We also have a series of security checks in place to ensure that participant responding is not fraudulent, including extra protections in Qualtrics and vetting of contact information. Random and/or fraudulent responders will be removed from subsequent analyses and will not be invited to participate in the remainder of the study protocol.

### Psychosis-Risk Measures

#### PQ

The positive items of the PQ will be administered to evaluate the frequency and distress of these symptoms in the past month [62,80]. Participants are instructed to endorse items only if not under the influence of drugs, alcohol or other medications while experiencing these symptoms. Endorsing ≥ 8 APPS has been found to have 90% sensitivity and 49% specificity with CHR status using the SIPS in a clinical population, and in undergraduate samples 13–18% met this criterion if 8 or more APPS were endorsed as distressing (this threshold will be used in the MAP study) [17–19].

#### Prime screen

The PRIME screen is a questionnaire similar in content and structure to positive symptom items within the SIPS [81]. The original author-recommended screening threshold (≥2 endorsements of “somewhat” or “definitely agree”, which will be used in the MAP study) to indicate a possible positive case yielded sensitivity of 0.90 and specificity of 1.00 with regard to SIPS CHR for psychosis diagnoses in a sample of 36 US adolescents and young adults referred for CHR evaluation [81] and sensitivity of 0.80, specificity of 0.48, and PPV of 0.52 in a sample of US adolescents and young adults receiving mental health services [14].

#### PROD-screen

The PROD-screen is a questionnaire assessing positive, negative, disorganized, general, and basic (e.g., subtle, self-experienced, self-reported deficits that often remain solely in the self-perception of the patient and do not show in behavior) symptoms present over the past year [64,82]. Although this questionnaire will be administered to all participants in the MAP study, the authors’ threshold of ≥2 symptoms was found to be too low in pilot testing, such that the majority of participants were meeting this threshold. In other studies, this threshold has been found to identify individuals with mild forms of delusions, hallucinations, or cognitive disturbances and has been found to have sensitivity of 0.80, specificity of 0.75, and PPV of 0.57 with regard to CHR status as determined by SIPS in a sample of 132 Finnish adults (mixed community/clinical sample) [82]. A self-report score of ≥2 yielded sensitivity of 1.00, specificity of 0.50, and PPV of 0.70 in a sample of Finnish adolescents who were referred for CHR evaluation [83]. This measure was chosen in part as it includes “basic” symptoms, symptoms that are qualitatively distinct from the other two psychosis screening measures in the MAP study, but as previously mentioned, in non-clinical samples we found very high rates of over endorsement of PROD items and removed it as part of the criteria for study entry. Although the PROD was not used for selection of participants for follow-up assessments, we will continue to use the PROD in our analyses to determine whether other psychosis-risk constructs, such as basic symptoms, improves identification of those at CHR for psychosis in combination with other questionnaires.

### Non-Psychotic Clinical Measures Associated with Psychosis

#### Dissociative experiences scale

The Dissociative Experience Scale assesses dissociative symptoms and experiences and is regarded as the gold standard for evaluating dissociation, demonstrating good reliability and validity, and has been associated with various psychosis outcomes [24,84]. Specifically, findings indicate a high test-retest reliability coefficient (0.84), high item agreement within the scale (Kendall coefficient = 0.70), and non-significant associations with theoretically unrelated variables (e.g., age) [84].

#### Center for epidemiologic studies—Depression scale

A shortened version of the Center for Epidemiologic Studies-Depression Scale will be used to assess the presence and severity of depressive symptoms in the past week. This scale has been found to be reliable and valid [85,86]. Reliability analyses suggest good internal consistency for the shortened scale [85], with a Cronbach’ s alpha of .76, high test retest reliability (0.9), and high sensitivity to identify depressive disorders (above 75%) [85,86].

#### State-trait anxiety inventory-trait form—anxiety subscale (STAI-trait)

The STAI-Trait [87] ascertains symptoms of generalized anxiety, with this version containing only items that load onto the anxiety factor, and excluding items loading on a depression factor. It has been found to be psychometrically sound [88,89]. Specifically, test-rest reliability for trait anxiety have been found to be high (0.73–0.86) and concurrent validity with other anxiety questionnaires also has been found to be high [87].

#### Social phobia scale (SPS)

The SPS is intended to measure social anxiety symptoms and has been found to be reliable and valid [90]. Specifically, the SPS has demonstrated high internal consistency (Cronbach’s α’s range from 0.89–0.94), high test-retest reliability (ranging from 0.91–0.93), and high discriminant validity (successfully discriminating between socially phobic and agoraphobic samples) [90].

#### General behavior inventory-patient version (GBI)

In order to assess manic and hypomanic symptoms, we will administer the GBI that asks participants to assess any observable elevations in their mood or behavior in the past year [91]. The GBI has been widely used in young adult populations [92,93], has been well validated and shows excellent psychometric properties, with fair to good positive predictive power (75–85%) and good to excellent negative predictive power (98–99%) [94]. The GBI also has been used to assess hypomanic symptoms in schizophrenia samples [95] and in psychosis-prone young adults [96,97].

#### The temporal experience of pleasure scale (TEPS)

The TEPS is a measure designed to index distinct trait dispositions to experience anticipatory pleasure (e.g., pleasure associated with expectation of reward) and consummatory pleasure (e.g., pleasure derived while engaged in an activity) [98]. The TEPS has exhibited good internal consistency (Cronbach’s alphas of 0.71–0.80) [98–100], high test-retest reliability [98,99], and strong construct and discriminant validity [98,99,101].

#### Pittsburgh sleep quality index (PSQI)

The PSQI is a self-report questionnaire that evaluates sleeping habits in the past month [102]. The component scores of the PSQI had an overall Cronbach’s alpha of 0.83, indicating a high degree of internal consistency [103] and have shown diagnostic sensitivity of 89.6% and specificity of 86.5% in distinguishing good and poor sleepers [103]. Sleep disruptions also have been associated with CHR for psychosis status at the Northwestern site using this measure [103].

#### Treatment history questionnaire

The Treatment History Questionnaire assesses past and current experiences with mental health services including therapy, medications, diagnoses, and hospitalizations, as well as whether, to what degree, and for what type of mental health issues participants are considering seeking treatment. The UMBC site created this questionnaire and collected data from over 400 undergraduate participants, with initial validity findings suggesting that students who reported current anxiety diagnoses had significantly elevated Beck Anxiety Index scores (means indicating “severe” anxiety) compared to non-endorsers and students who reported current depression diagnoses had significantly elevated Beck Depression Inventory-II scores (means indicating “moderate” depression) compared to non-endorsers.

### Risk Factor Measures

#### Family history of mental disorders

The screening questions from the Family Interview for Genetic Studies [104] were adapted into an inventory to get a general sense of family history of mental disorders, which has been used at the TU site and has been associated with one psychosis-risk screening tools, the PQ, (see Risk factors potentially associated with CHR for psychosis in non-clinical populations).

#### Sociodemographic questionnaire

This questionnaire is based on the face sheet of the Cross Racial Identity Scale (particularly relevant for ethnically diverse populations) with additional questions added [105]. Demographic information including gender, age, ethnic background (including detailed information on immigrant status and country(ies) of origin of self/family members), race, importance of religion, type of religion, family income, family socioeconomic status, parental occupation, neighborhood characteristics from childhood (e.g., ethnicity/population densities from childhood), academic status/performance, current employment, and information on developmental milestones will be assessed. In addition, participants will provide the ZIP code of the neighborhood in which they were raised/current ZIP code as validation tools for neighborhood characteristic questions (i.e., to geocode neighborhood-level characteristics per participant).

#### Life events checklist (LEC)

The LEC assesses exposure to discrete traumatic life events (TLEs) [106,107], with an “other” category that will be supplemented with being severely bullied during childhood and adolescence, given the associations between bullying and psychotic symptoms [108]. Rates of TLEs are particularly high in psychosis samples compared to non-psychiatric controls and other clinical samples (e.g., depression, substance abuse) [109]. The LEC has been shown to have good convergent validity with well-established measures of trauma histories, such as The Traumatic Life Events Questionnaire, and moderate temporal stability [107]. Specifically, test-retest reliability has been found to be high (with Kappa = 0.61 and retest correlation = 0.82), has been significantly correlated with similar trauma and life event measures, and has been significantly correlated with psychopathology associated with traumatic life events [107].

#### The childhood trauma questionnaire short-form (CTQ)

The CTQ is a self-report inventory for ages 12+ that taps into 5 types of maltreatment: emotional, physical and sexual abuse, and emotional and physical neglect [110,111]. The CTQ is the most widely used self-report measure of trauma exposure in the psychosis literature and also has strong reliability and validity [110,111]. Specifically, the CTQ has been found to have high internal consistency, ranging from 0.79 to 0.94, good test-retest reliability over a 2- to 6-month interval (intraclass correlation = 0.88), as well as convergence with the Childhood Trauma Interview [110].

#### Perceived stress scale (PSS)

The PSS is a measure of perceived global stress and coping ability that focuses on the predictability and controllability of events in the past month [112]. This scale has established high concurrent and predictive validity with physical outcomes (e.g., colds), psychiatric outcomes (e.g., depression, social anxiety), and with other measures of stress such as negative affect and impact of life event scales, as well as moderate internal and test-retest reliability (α = 0.89) [113–117]. Higher PSS scores have discriminated between those at psychosis populations and non-psychiatric control samples, and have been associated with schizotypy [116–118].

#### Experiences of discrimination instrument

This measure assesses participants’ reactions to being treated unfairly and types of situations in which the participant may have experienced discrimination based on race, ethnicity, or color. Frequency of these situations/reactions also is assessed [119]. The measure has been psychometrically validated, showing good reliability and validity [119], and we already have found that this scale is related to APPS in a young adult sample [120]. This scale also has shown good internal consistency (Cronbach’s α = 0.74 or higher), high test-retest reliability (correlations = 0.69 or higher), and high concordance between respondents and key informants (68%) [119].

#### Drug use frequency

The Drug Use Frequency measures frequency of use of various substances and has been established to have adequate reliability and validity [121]. Specifically, this measure shows high concordance with similar measures (percent agreement between 0.83–0.98) and significant correlations (moderate to good agreement) with partner collateral reports [121]. Additional questions were added to this measure based on relevant psychosis studies, as well as preliminary data from UMBC and TU sites. Specifically, information on setting of substance use (i.e., alone or with others), whether the substance use interferes with various areas of functioning, age at first use, estimations of lifetime frequency, and period of last use (e.g., >1 year ago).

#### Social functioning scale (SFS)

The SFS is a scale normed on a sample of individuals with schizophrenia and non-psychiatric controls from the general population [122]. It assesses functioning in social engagement, interpersonal contact, recreation, competence in independent activities, and engagement in daily activity/occupation. It has high internal reliability and consistency, with strong discriminative power [122]. Specifically, internal consistency has been shown to be high (Cronbach’s α = 0.80), inter-rater reliability with collateral reports of social functioning also have been found to be high (Cronbach’s α = 0.94), and this scale can distinguish between schizophrenia patients and non-psychiatric control participants [122]. In addition, we have previously found significant associations between the SFS in and the PQ, suggesting that it is an appropriate measure to use when examining subthreshold psychotic symptoms [123].

#### The quality of life enjoyment and satisfaction questionnaire—Short form

This is a questionnaire assessing enjoyment and satisfaction with several areas of daily functioning within the past week [124]. Test-retest reliability ranged from 0.63 to 0.89 and internal consistency ranged from 0.90 to 0.96 in a validation sample of adults with depression receiving outpatient care [124].

#### Lubben social network scale-revised

The Lubben Social Network Scale-Revised is a questionnaire developed to capture social support by measuring the size, perceived closeness, and frequency of contacts of a respondent’s social network across two subscales (family and friendships) [125]. Cronbach’s alphas have been found to range from 0.78 [125] to 0.90 [126]. Similarly, among undergraduates at UMBC, Cronbach’s alphas ranged from 0.78 to 0.79 (unpublished UMBC data).

### Semi-Structured Interviews (T2)

#### Structured interview for psychosis-risk syndromes (SIPS) version 5.6

The SIPS is the most commonly used interview in the US for assessing psychosis-risk syndromes and has established predictive validity of conversion to psychosis, specificity, and inter-rater reliability [9,11,12,127,128]. Participants will be deemed at CHR for psychosis by meeting criteria for one or more (out of 3) of the psychosis-risk syndromes. Low clinical risk status will be defined as not meeting criteria for any of the psychosis-risk syndromes. We also will examine the DSM-5 attenuated psychosis syndrome (assessed through the SIPS) and other SIPS risk syndromes (e.g., persistence) in supplementary analyses. The Negative Symptom Inventory—Psychosis Risk (NSI-PR) also was administered to assess the full range of negative symptoms, as this interview as developed specifically for CHR sample [129].

#### The structured clinical interview for DSM-5, research version (SCID)

Presence of DSM-5 mental diagnoses will be determined using the SCID [130], which has demonstrated moderate-to-strong reliability and is the “gold standard” in determining the accuracy of clinical diagnoses [131].

#### Semi-structured interviews training and reliability

Interviewers are advanced level clinical doctoral students, postdoctoral employees, and advanced level research assistants (with at least a bachelor’s degree and 2–3 years of experience working with clinical populations). Training and reliability will involve multiple procedures in order to ensure accurate and consistent diagnoses across the study sites. It should be noted that the 3 PIs (and most of their staff) all have extensive training and expertise with the SIPS and the SCID, with over 17 years of experience with these measures for each PI. The training/reliability procedures will be as follows:

1. All study PIs and clinical interviewers will attend a Gold Standard SIPS training at TU at the MAP study and every year of the MAP study for new staff (note: this is the same procedure employed by the NAPLS consortium). This training (which will be a repeat training for many) will ensure consistency in ratings across the site interviewers. All interviewers earn a SIPS certification at the end of this training (one of the PIs is a Yale certified SIPS trainer).
2. Following SIPS training, each interviewer will be required to rate 3 SIPS and 3 SCID videos from previous studies, which the PIs have rated and established agreed upon ratings. Interviewers will be required to reach inter-rater reliability of Kappa ≥0.80 for all ratings prior to conducting interviews. In addition, each interviewer will be required to observe a minimum of 2 SIPS/SCID interviews, followed by being observed conducting a minimum of 2 interviews by an experienced interviewer. Each PI will be responsible for ensuring proper implementation of the aforementioned procedures. Interviewers will be blind to participants’ potential psychosis risk status (i.e., QHR or QLR).
3. Following completion of interviews, a narrative will be written by the interviewer with a detailed explanation of the ratings that contributed to risk syndrome and DSM-5 diagnoses, available prior to consensus diagnostic meetings.
4. Consensus review will involve 1. Consensus diagnostic phone meetings held twice a week by 3 experienced interviewers (one from each site) to independently review the diagnostic material (decisions will be made by majority vote). The clinician-raters will join these conference calls to discuss specific interviews. More complicated cases will be triaged to the PIs of the study for additional consensus calls and the PIs will alternate reviewing the less complicated decisions bi-weekly to ensure agreement with decisions. Any disagreement by a PI after reviewing cases will be addressed in the PI consensus call and 2. The PIs will have bi-weekly consensus calls to review more complicated cases, as well as disagreements in the initial stage of review. Decisions will be decided based on a majority vote among the 3 PIs.
5. Reliability will be assessed by randomly selecting 10% of interviews across the sites and coding interviews based on video recordings every 6 months by study interviewers. Kappa scores of 0.80 or higher will be judged reliable. If Kappa scores fall below 0.80, discrepancies will be examined and discussed among the PIs and all study interviewers to address potential drift and site differences.

### Brief Computerized Neuropsychological Battery & IQ Estimate (T2)

For exploratory purposes, to ascertain if there are cognitive differences between the study sites and to determine whether cognitive performance is similarly predictive of CHR status in non-clinical samples compared to clinical samples, we will administer the Wechsler Abbreviated Scale of Intelligence-II subscales vocabulary and matrix reasoning (to ascertain an IQ estimate [132]) and the University of Pennsylvania Computerized Neuropsychological Testing battery [133]. It should be noted that the aims of the MAP study do not include assessment of cognitive functioning; however, given the novelty of the sample, neuropsychological characterization of these participants could potentially influence future CHR identification and treatment designs.

## HYPOTHESES

Based on our preliminary data (see Criterion Validity), we anticipate that approximately 186 participants (18.6% of 1000) of the QHR group who are interviewed will be classified as CHR by the SIPS. While the analyses used in the MAP study are data-driven, based on our preliminary data (see Criterion Validity), we anticipate that higher unusual thought content, a family history of major mental disorders, and higher levels of anxiety and depression will be important variables in our final questionnaire. Although the prediction of CHR for psychosis likely only partially overlaps with prediction to conversion to psychosis, “risk calculators” for conversion to psychosis have determined that decreases in social functioning contribute to improved prediction of conversion [21], which likely will be the case in the proposed study. Nevertheless, every questionnaire included in the proposed project has been linked to risk for psychosis in our preliminary data and published CHR studies; which supports the likelihood that additional meaningful latent classes will emerge, as was the case in our preliminary findings (see Inclusion of Non-APPS Clinical Measures, Inclusion of Risk Factor-Based Measures & Preliminary Studies).

### Analytic Plan

The following section sets forth an analytic plan based on the specific aims of the study. It should be noted that the present study has assembled an analytic team of researchers who are established experts in the methods described below. All analyses described below will be conducted in SAS version 9.4 (Cary, NC, USA), Mplus version 7.4 (Los Angeles, CA, USA), and R version 3.1.1 (Vienna, Austria).

### To Determine Norms and Prevalence Rates of APPS across 3 Diverse Sites

The proposed study aims to determine commonalities among responses to APPS using 3 well-known questionnaires across 3 sites, as well as APPS prevalence rates and norms for adolescents/young adults in the US (using all 12,000 participants). We also aim to identify demographic variability in APPS across sites. Initial analyses will focus on descriptive characteristics of item and total scale scores for APPS from the 3 psychosis-risk screening measures. Item-based analyses will (1) determine prevalence rates for each APPS (i.e., rates of endorsement of each question) for each scale, which will allow us to descriptively identify items that are endorsed at high rates, but may not be clinically-relevant (tested in #2 below), and (2) determine demographic and site differences in prevalence rates at the symptom level (i.e., by age, gender, and race/ethnicity) with simple bivariate analyses. We also will use previously established cut-off scores for the three self-report screening measures to descriptively determine if prevalence rates above these cut-offs are similar in non-clinical samples compared to published clinical samples (in addition to determining whether these prevalence rates differ by site and demographic factors). Frequency of item endorsement across sites and demographic categories (e.g., race, gender) will be examined using chi-square tests, and total scale score differences will be examined using analysis of variance.

While not part of Aim 1, as an auxiliary analysis we also will employ item response theory (IRT) to determine the items that best tap into APPS across the 3 screening measures, which is the best method for this type of analysis [134,135]. We will evaluate unidimensionality of the construct by using both exploratory factor analysis (EFA) and confirmatory factor analysis (CFA) procedures. In the EFA, unidimensionality will be supported if the ratio of the first to second eigenvalue exceeds 4 [136,137]. We will confirm unidimensionality using CFA and will evaluate a one-factor model using multiple fit indices, including the Comparative Fit Index (>0.90) and the root mean square error of approximation (RMSEA; <0.05 and upper limit of 90% CI < 0.08). IRT parameters may demonstrate differential item functioning (DIF), which suggests that items may provide different information across different identifiable individuals. We will probe DIF as a function of demographic characteristics and site of participation using model-based log-likelihood difference tests. This latter analysis will provide information about whether the APPS construct is psychometrically equivalent across sites and participant characteristics. If item pools do not satisfy these criteria, we will examine alternative structures, including multidimensional IRT models [134,138], that may provide a better account for the data [139]. If necessary, these models will be examined by fitting exploratory bifactor models [140] to the data (using the bigeomin rotation [141]), and we will rely on traditional goodness-of-fit indices for these models to identify well-fitting models. Test information function also will be examined to assess whether to keep or eliminate questionnaire items.

### To Develop a Screening Questionnaire That Is Validated against the SIPS in Order to Identify Those at CHR for Psychosis Using Both Symptom-Based and Risk-Factor Based Measures (QHR and QLR Participants *n* = 2000)

First, we will use LCA to identify distinct profiles of symptoms and risk factors [142]. The optimal solution will be identified using the Bayesian Information Criterion (BIC) and bootstrap likelihood ratio test (BLRT) [143]. The BIC is a relative fit index that permits comparisons across model solutions. The BLRT examines improvement in fit when models increase in the number of classes by one. Comparisons of solutions across recruitment sites and/or sampling methodology will be conducted using multiple group latent class models using log-likelihood difference tests between models with and without equality constraints across model parameters. Second, we will use the LCA profiles to distinguish between classes predominantly inclusive of individuals at CHR status versus classes without individuals at CHR status. To reduce the number of items for the resultant screening tool, the LCA results will identify the specific scales that discriminate between CHR-risk classes. Those discriminating scales will then be subjected to IRT analyses on a scale-by-scale basis. These analyses will be conducted to remove redundant items (identified by difficulty and discrimination parameters [144] and model-based tests of total information) from each scale. However, we will be mindful to not eliminate items that are highly relevant to the substantive construct at hand. Critically, these analyses will be conducted on the initial sample of 12,000 to maximize reliability of the results.

Following LCA, recursive partitioning (RP) primarily will be used to obtain cut-off scores for scales/items included in the final questionnaire. While some of the variables entered into RP will be summed scaled scores, these summed scores will be entered after IRT analyses to reduce the number of items. RP is a nonparametric exploratory data mining procedure that builds a “tree” to identify exhaustive orthogonal subgroups of a population whose members share common characteristics that influence the dependent variable [145,146]. First, items and summed scores will be entered from each class defined in the LCA (in separate models for each class) to determine cut-off scores and appropriate items for each class. In RP, sample participants are split into smaller and smaller groups using a recursive procedure until each group is perfectly homogenous. RP also will provide cut-off scores for each variable for each split of the tree, which will allow for proper development of a scale. The strength of this method is that it detects and describes complex interactions that identify a parsimonious subset of independent variables that is sufficient to predict the specified outcome variable, in this case, CHR for psychosis. The second stage of the RP procedure consists of using cross validation to prune the full tree to an “appropriate size” to reach this goal (cross-validation procedures described below), which consists of repeated re-estimation of the RP tree using random samples (with replacement) from the original dataset. The cross-validation procedure estimates the prediction error, and related standard error, for each of the splits in order to improve standard errors. Appropriate tree size is usually determined by cost, model complexity, or cross validation. Breiman and colleagues [147] suggested using a ± 1 standard error rule to choose appropriate tree size. Hence, a good-fitting tree is one with the least number of splits and the smallest cross-validation error, given that the tree’s cross-validation error plus its standard error is less than 1, as 1 is equal to the relative error of a model with no splits, or guessing. We will then determine sensitivity, specificity, PPV, and negative predictive value (NPV) for each subgroup identified with RP.

**Figure 3.**
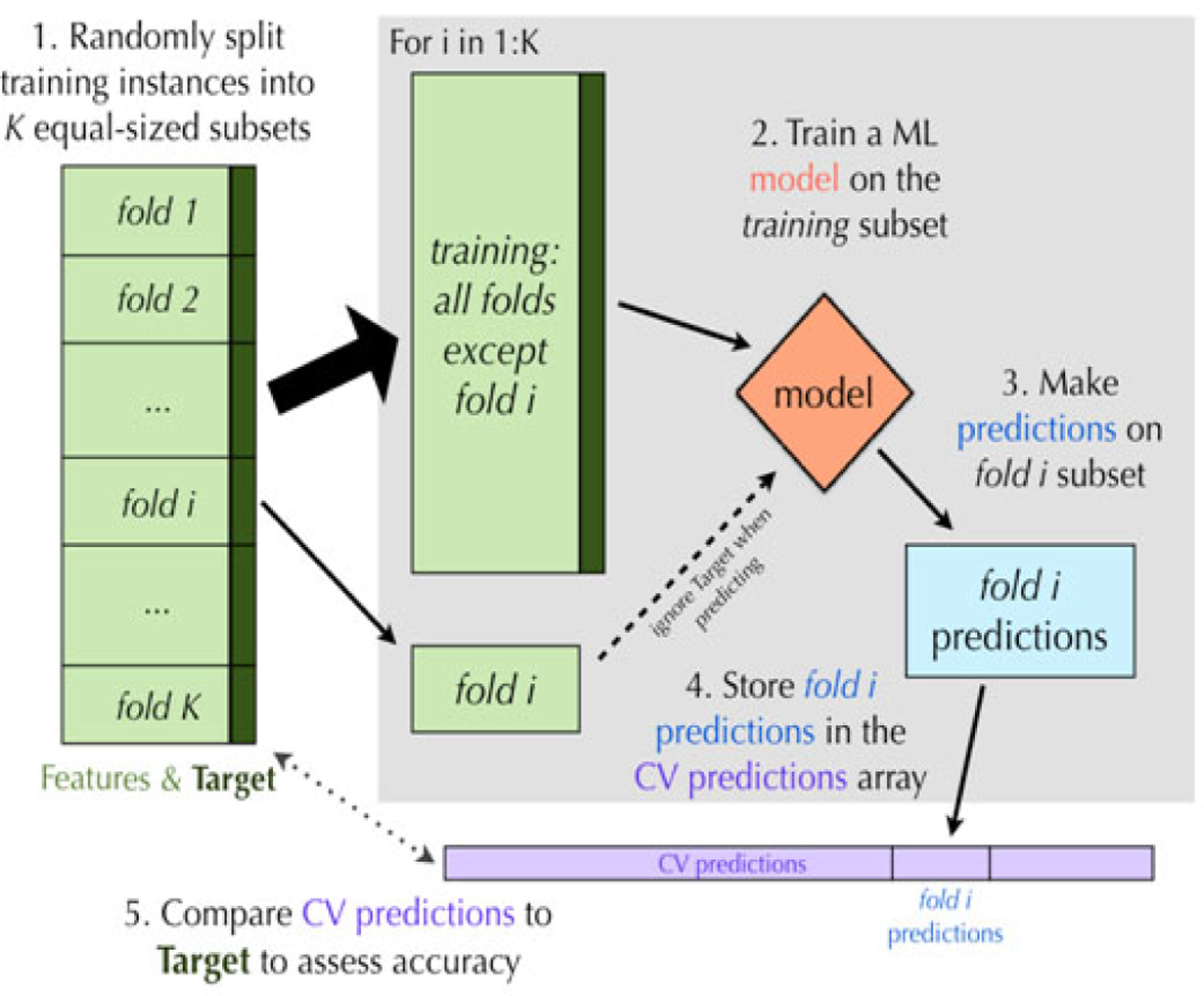
K-fold cross-validations.

For the analyses described above, the final questionnaire will include subgrouped items, potentially including summed scores from reduced scales, which have between a 40–60% PPV for CHR for psychosis, representing a substantial improvement over existing measures. Given that the proposed questionnaires take approximately 45 min to complete, the goal for a maximum completion time of 20 min for the final questionnaire appears achievable using the above steps. The final questionnaire, as well as the PPV, area under the curve (AUC), and accuracy of the final model, will be cross-validated using k-fold cross-validation, in this case 5-fold [148–150]. With 5-fold cross-validation, the data are randomly split into 5 subsets. The complete questionnaire development approach is applied to 4/5 of the data, the “training set,” with the remaining 1/5 used for validation (the “testing set”). In this case, the BIC will be assessed for the 1/5 validation data. This is repeated for each of the 5 validation data sets. The average BIC across all 5 trials is computed, as well as the variation of the BIC measures, with higher BIC variability indicating an overfit model for the selection of the questionnaire items. Thus, the variance of the metric (BIC, AUC, PPV, accuracy) across the 5 runs is as important as the mean, where a good model should show similar performance across all the splits. The mean and variability of the 5 PPV, AUC, and accuracy measures for the 5 validation data sets will then be computed to assess the questionnaire. A graphic illustrating k-fold cross-validation is provided in Figure 3. At each step, accuracy, BIC, AUC, and PPV, will be calculated for each of the 5 folds, with the average and variance of each of these statistics calculated over the folds. Omega values and other metrics of reliability will be calculated to assess reliability of the final questionnaire. All analyses will be conducted for the progressive syndromes on the SIPS and the DSM-5 attenuated psychosis syndrome; additional SIPS syndromes (e.g., persistence) will be examined in auxiliary analyses.

Also, once data collection is complete, we will re-evaluate the statistical analyses plan to include any new methods that may be more appropriate for our primary aims (e.g., network models).

### Secondary Analyses

As a secondary analysis, we will conduct receiver operating curve (ROC) analyses for each psychosis-risk screening measure to determine whether previously established cut-off scores from clinically based samples correspond to cut-off scores from non-clinical samples compared against the SIPS. Although our main aim is to utilize measures that tap into APPS, as well as other domains, it remains critical to determine whether the existing cut-off scores from psychosis-risk screening measures are applicable to non-clinical populations. ROC curves plot the true positive rate against the false positive rate for the different possible cut-points of a measure, such that any increase in sensitivity likely will be accompanied by a decrease in specificity. Statistical significance is determined when the confidence interval for the AUC is greater than 0.50, indicating that the test predicts diagnosis better than chance [151]. For exploratory purposes, we also will determine whether non-psychosis symptoms, risk factors, and neurocognitive functioning is similar among a non-clinical CHR sample compared to published clinical CHR samples.

#### Exploratory analyses

For exploratory analyses, we will examine the role of gender. Gender will be evaluated as a moderator of the relationship between class membership and CHR status. Finally, we will examine consistency in the general pattern of results in the LCA, data reduction procedures, and development of short-form screening measures across sexes by stratifying our sample by gender. While the primary aims of the MAP study are to develop a screening tool that is effective regardless of gender, given the potential for gender differences in responding to questionnaires, we will create separate screening tools for males and females if stratifying by gender substantially improves PPV, AUC, and measures of accuracy.

#### Managing missing data

We will examine the pattern of missing data using Littleʼs Missing Completely At Random MCAR test [152]. We anticipate low enough levels of missing data (based on our studies) and that we will find a non-significant MCAR test, which would indicate that full information maximum likelihood (FIML) estimation will be appropriate for the LCA and IRT analyses. Thus, we plan to include all participants in those analyses. Further, the DFA will also be conducted with 5-fold replication.

### Power Considerations

For parametric statistical tests (based on GPower Software), power estimates are based on alpha = 0.05 and identification of small effect sizes (i.e., *d* = 0.20; *r* = 0.10; OR = 1.5). Where noted below, we consider corrections to alpha based on multiple tests. Before analyses are conducted, distributions of variables will be examined to identify non-normality in variables. Analyses that examine non-normal dependent variables will rely on robust maximum likelihood estimation methods that correct analyses for violations of non-normality, while retaining the raw distribution of the variables or transform the variable depending on the variable’s distribution. Aim 1 emphasizes the description of and comparisons between individual items and total scale scores on demographic characteristics and site of participation using the full self-report sample (*n* = 12,000). Comparisons across racial groups and participation sites for self-report measures all have sufficient power (>0.95) after correcting for multiple comparisons using a corrected p-value of .008 (the most conservative value estimable for this type of analysis in GPower). As an auxiliary analysis to Aim 1, the underlying dimension of APPS across all three psychosis-risk screening measures using IRT (or similar methods, if unidimensionality is not met) also will be examined. Recommendations for implementing IRT-based analyses include having at least 700 participants or having at least 100 participants per response option for the measure with the greatest number of response options [134,153]. The present sampling design satisfies both of these conditions. Power for detecting changes in model fit (DIF) by demographics (e.g., gender) and other variables (e.g., site) exceeds 0.95 (R software package). Aim 2 will develop a self-report questionnaire that will be useful in predicting CHR status in a non-clinical sample. There are no established means of estimating power for LCA. However, recommendations in the literature suggest that there are often concerns about identified classes being too small, often described as representing less than 5% of the sample. In our sample, a class of 5% would still include 600 (if based on the 12,000 total) or 100 (if based on the interview sample only, *n* = 2000). Thus, there is sufficient power to detect differences between classes—published reports with clinically meaningful results have relied on samples with as few as 200 cases. Further, RP analyses, PPVs, NPVs, sensitivity and specify are not constrained by sample size; therefore, there is sufficient power for these analyses [147]. Finally, power estimates suggest that our planned examination of gender differences is feasible.

## REPRODUCIBILITY AND RIGOR

The MAP study has taken numerous steps to ensure reproducibility and rigor of results including 1. Ensuring adequate power for the main study aims; 2. A detailed statistical plan (with expertise relevant to this plan); 3. Recruitment of a community-based sample across multiple suburban, rural, and urban areas with variability in anticipated P characteristics across sites (e.g., variations in rates of substance use, treatment seeking, and prodromal symptoms among participants), which should increase the generalizability of our findings, produce prevalence rates of important risk factors that are sufficient for proposed analyses—ultimately, variability in P characteristics is necessary to improve precision and reduce variability in our proposed findings; 4. Inclusion and exclusion criteria are based on known risk periods for psychosis, including consideration of different ages of onset for males and females; 5. Careful procedures are in place for blind and independent assessment of the main study variables; 6. An appropriate plan for handling of missing data; and 6. A sophisticated cross-validation procedure to ensure replicability of findings.

## STUDY LIMITATIONS

The MAP study is somewhat limited by constraints of self-reported data, as our preliminary findings and findings from others have demonstrated that questionnaires can be limited by a number of factors, such as participants’ ability to comprehend questions and potential differences in participants’ reading ability and other individual-level factors that make influence responding. One benefit of the MAP study is our ability to determine 1. If there are questions that are over-endorsed by participants (potentially indicating lack of comprehension, which we have found in our pilot studies), and 2. If cognitive factors (or other individual-level factors) influence responding to specific items. Reliance on self-report data also comes with potential difficulties due to lack information about how cultural context influences responding. For instance, findings suggest that race, age, and gender influence responding on psychosis-risk questionnaires [154–157]. Part of the advantages of this study will be an ability to determine whether a variety of factors influence questionnaire responding (e.g., cognitive functioning, gender, race, ethnicity, etc.), which we can incorporate into the final questionnaire. In addition, there always is a possibility that the questionnaire produced by this study will be used (or misused) in lieu of clinical interviews. Although a disclaimer will be included with the questionnaire stating that it is not meant to replace clinical diagnoses and should be used as a first step to determine whether in person assessment is necessary, any psychosis-risk screening through questionnaires comes with potential limitations. While limitations of questionnaires will inherently be present, we will be well-suited to explore prominent concerns relevant to these types of studies.

## SUMMARY OF THE MAP STUDY

The MAP study will be the first to develop a valid psychosis-risk screening measure (both a long and short version) using non-clinical adolescent/young adult populations in the US, incorporating both clinical and risk-factor based measures. Additionally, this will be the first study to establish norms for APPS in the US among adolescents/young adults during the highest risk period for the onset of psychotic symptoms. The MAP study has major public health and mental health implications, as it has the ability to dramatically improve identification of those at risk for psychosis, which is critically important as duration of untreated psychosis has been associated with a worsened course of psychotic disorders.

## Figures and Tables

**Table 1.** Prediction of CHR using questionnaires.

Variable permutations	Sensitivity	Specificity	PPV	NPV
1. PQ cut-off alone	86.7	46.2	18.6	96.1
2. PQ + Broad Fhx	86.7	63.2	25.0	97.1
3. UTC + LCA Clinical Symptoms	80.0	66.0	25.0	95.9
4. UTC + Broad Fhx	86.7	69.8	28.9	97.4

**Table 2.** Anticipated distribution of gender and race by site.

Race Categories	Greater Philadelphia, PA	Greater Chicago, IL	Greater Baltimore, MD
% female	51.9%	51.6%	52.8%
American Indian/ Alaska Native	0.2%	0.5%	0.3%
Asian	5.4%	7.0%	3.9%
Native Hawaiian or Other Pacific Islander	<0.1%	0.04%	0.04%
Black or African American	21.7%	27.7%	43.2%
White	63.5%	62.0%	50.2%
More Than One Race	1.6%	2.8%	2.3%

## ABBREVIATIONS

SIPS: Structured Interview for Psychosis-Risk Syndromes
PPVs: positive predictive values
CHR: clinical high risk
APPS: attenuated positive psychotic symptoms
MAP: Multi-site Assessment of Psychosis-risk study
APS: Attenuated Psychosis Syndrome
PQ: Prodromal Questionnaire
PQ-B: Prodromal Questionnaire-Brief
SPS: Social Phobia Scale
LCA: latent class analysis
Fhx: familial high risk variable
UTC: unusual thought content
QHR: Questionnaire High Risk
QLR: Questionnaire Low Risk
T1: Time 1
T2: Time 2
PROD: Prod-screen
STAI: State-Trait Anxiety Inventory
GBI: General Behavior Inventory-Patient Version
TEPS: The Temporal Experience of Pleasure Scale
PSQI: Pittsburgh Sleep Quality Index
TRHQ: Treatment History Questionnaire
LEC: Life Events Checklist
TLEs: traumatic life events
CTQ: The Childhood Trauma Questionnaire Short-Form
PSS: Perceived Stress Scale
SFS: Social Functioning Scale
SCID: The Structured Clinical Interview for DSM-5, Research Version
IRT: item response theory
EFA: exploratory factor analysis
CFA: confirmatory factor analysis
RMSEA: root mean square error of approximation
DIF: differential item functioning
BIC: Bayesian Information Criterion
BLRT: bootstrap likelihood ratio test
RP: recursive partitioning
NPV: Negative predictive value
AUC: area under the curve
ROC: receiver operating curve
MCAR: Missing Completely At Random
FIML: full information maximum likelihood
DFA: discriminant function analysis
